# Health Care Services Utilization and Health-Related Quality of Life of Syrian Refugees with Post-Traumatic Stress Symptoms in Germany (the Sanadak Trial)

**DOI:** 10.3390/ijerph18073408

**Published:** 2021-03-25

**Authors:** Thomas Grochtdreis, Susanne Röhr, Franziska U. Jung, Michaela Nagl, Anna Renner, Anette Kersting, Steffi G. Riedel-Heller, Hans-Helmut König, Judith Dams

**Affiliations:** 1 Department of Health Economics and Health Services Research, Hamburg Center for Health Economics, University Medical Center Hamburg-Eppendorf, Martinistr. 52, 20246 Hamburg, Germany; h.koenig@uke.de (H.-H.K.); j.dams@uke.de (J.D.); 2 Institute of Social Medicine, Occupational Health and Public Health (ISAP), Medical Faculty, University of Leipzig, 04103 Leipzig, Germany; susanne.roehr@medizin.uni-leipzig.de (S.R.); franziska.jung@medizin.uni-leipzig.de (F.U.J.); steffi.riedel-heller@medizin.uni-leipzig.de (S.G.R.-H.); 3 Department of Psychosomatic Medicine and Psychotherapy, University Medical Center Leipzig, 04103 Leipzig, Germany; michaela.nagl@medizin.uni-leipzig.de (M.N.); anna.renner@medizin.uni-leipzig.de (A.R.); anette.kersting@medizin.uni-leipzig.de (A.K.)

**Keywords:** post-traumatic stress, mental health, health care services utilization, health care costs, health-related quality of life, Syrian refugees

## Abstract

Refugees who have fled from the ongoing civil war in Syria that arrived in Germany often develop post-traumatic stress symptoms (PTSS). The aim of this study was to determine health care services utilization (HCSU), health care costs and health-related quality of life (HrQoL) of Syrian refugees with mild to moderate PTSS without current treatment in Germany. The study was based on the baseline sample of a randomized controlled trial of a self-help app for Syrian refugees with PTSS (*n* = 133). HCSU and HrQoL based on the EQ-5D-5L and its visual analogue scale (EQ-VAS) were assessed with standardized interviews. Annual health care costs were calculated using extrapolated four-month HCSU and standardized unit costs. Associations between health care costs, HrQoL and PTSS severity were examined using generalized linear models. Overall, 85.0% of the sample utilized health care services within four months. The mean total annual health care costs were EUR 1920 per person. PTSS severity was not associated with health care costs. The EQ-5D-5L index score and the EQ-VAS score was 0.82 and 73.6, respectively. For Syrian refugees with higher PTSS severity, the EQ-5D-5L index score was lower (−0.17; *p* < 0.001). The HCSU and the resulting health care costs of Syrian refugees with mild to moderate PTSS without current treatment are low and those with a higher PTSS severity had a lower HrQoL.

## 1. Introduction

Since the beginning of the civil war in Syria in 2011, the share of Syrians in persons that applied for international protection in Germany to escape from the ongoing civil war has been high. Between 2014 and 2019, almost 2 million asylum seekers arrived in Germany [1]. Of all asylum seekers and refugees in Germany in 2019, Syrians made up the largest proportion, with 31% [2]. Before and during the flight, refugees are confronted with significant traumatizing situations, such as war, terror and life-threatening situations to themselves and relatives [3]. After their arrival in Germany, a large majority of Syrian refugees reported having personally experienced and/or witnessed traumatizing situations, accompanied by a high prevalence of post-traumatic stress symptoms (PTSS) [4]. In general, asylum applicants and refugees have a high risk of mental disorders with relation to trauma and stress, but also affective disorders as well as schizophrenia, schizotypal disorders and delusional disorders [5,6,7,8,9,10,11]. In a study analyzing mental distress among Syrian refugees who have been staying in Germany since 2014, it was found that 11.4% of all participants met the diagnostic criteria for post-traumatic stress disorder (PTSD) within 6 months after arrival in Germany [4]. By contrast, in the general population of the European Union, the 12-month prevalence of PTSD was estimated to be just about 1% to 3%, with about 8 million persons affected [12].

Characteristic PTSS are persistent intrusion symptoms and avoidance of stimuli, negative alterations in cognitions and mood, marked alterations in arousal and reactivity associated with the traumatic event, causing clinically significant distress or impairment in social, occupational or other important areas of functioning [13]. In addition to comorbid mental health disorders, PTSD is associated with poorer physical and mental health-related quality of life (HrQoL) and general health complaints [14,15,16]. For refugees with comorbid PTSD and depression that arrived in Switzerland, a statistically significantly lower mental and physical HrQoL compared with refugees without diagnosis was found [17]. According to a recently published analysis of survey data, the mental and physical HrQoL of Syrian asylum applicants and refugees was lower compared to that of a German normative sample [18]. In addition to the country of origin, HrQoL of asylum applicants and refugees was also determined by sex, age, education, employment and marital status [3,18,19].

The annual economic costs of PTSD were on average more than EUR 1000 per affected European person and EUR 8.3 billion in Europe in total [20]. According to a systematic review of cost analyses in PTSD, the mean annual health care costs of PTSD even ranged from EUR 9591 to 14,802 per affected person in Germany [21]. Nevertheless, in terms of health care services utilization (HCSU) of recently arrived refugees in Germany, no comprehensive analysis has been published so far. A systematic review of HCSU of people with a migrant background in Germany concluded that persons with a migrant background utilized health care less often than persons without a migrant background [22]. Likewise, an exploratory study analyzing disparities in access to health care among asylum seekers in Germany found lower HCSU with respect to primary care physicians and specialist outpatient physician services, whereas inpatient care was utilized more often [23]. For asylum seekers in Germany, associations between worse health status and higher utilization of primary care physicians, specialist outpatient physician services and inpatient care have been identified [24].

In order to be able to identify starting points for providing cost-effective health care services targeted at Syrian refugees with untreated PTSS, it is necessary to describe the health economic consequences of PTSS with respect to HCSU, health care costs and HrQoL [25]. To our knowledge, those consequences have not been analyzed yet for Syrian refugees with PTSS that arrived in Germany during the European migrant and refugee crisis. Therefore, the aim of this study was to evaluate the economic consequences of PTSS with respect to HCSU and costs from the health care payers’ perspective, and HrQoL for Syrian refugees with mild to moderate PTSS without current psychotherapeutic treatment in Germany based on the “Sanadak” trial [26,27].

## 2. Materials and Methods

### 2.1. Sample

The study was based on the baseline sample (N = 133) of a randomized controlled trial to measure the effectiveness and cost-effectiveness of a self-help app for Syrian refugees with PTSS (the “Sanadak” trial) [26,27]. The optimal sample size for estimating a significant treatment effect of N = 140 was calculated given a moderate between-group effect at 3 months after the intervention (Cohen’s d = 0.50), a significance level of α = 0.05 and a statistical power of 1 − β = 0.90. Study participants were recruited in the urban areas of Leipzig and Dresden and Halle/Saale, Germany, between October 2018 and December 2019. Detailed recruitment strategies as well as the flowchart of sample selection can be found elsewhere [27,28,29].

Inclusion criteria were being aged 18 to 65 years, having experienced at least one traumatic event and subsequent mild to moderate PTSS severity measured by the Posttraumatic Diagnostic Scale for DSM-5 (PDS-5 ≥ 11) [30]. Persons were excluded if they lacked a compatible device (Android/iOS) for the self-help app, had severe PTSS (PDS-5 ≥ 60), severe depressive symptomatology, acute suicidal tendencies, if they were currently in outpatient psychotherapeutic treatment/psychiatric treatment and/or received psychopharmacological medication or were pregnant. Screening for eligibility was carried out by trained native Arabic-speaking study nurses. In total, 170 persons were screened for eligibility, and of those, 32 persons did not meet inclusion criteria (19%) and 4 persons declined further participation (2%). Of those that did not meet inclusion criteria, 12 persons did not have mild to moderate PTSS severity (7% of all persons screened), 5 persons were currently in outpatient psychotherapeutic treatment (3%), 3 persons had severe depressive symptoms (2%) and 2 persons were pregnant (1%) [27,29].

The “Sanadak” trial was approved by the Ethics committee of the Medical Faculty of the University of Leipzig, Germany (ID: 111-17-ek) and was conducted in accordance with the Declaration of Helsinki. The trial was registered prospectively in the German Clinical Trials Register (registration ID: DRKS00013782). Written informed consent was obtained from all study participants after confirmation of participation. Details regarding the study design, and the results in clinical outcomes of the “Sanadak” trial can be found elsewhere [26,27].

### 2.2. Measures

Participants were interviewed by trained native Arabic-speaking study nurses using a written, structured questionnaire (paper-and-pencil assessment, face-to-face). All instruments used were available in Arabic language or were translated into Arabic [31].

HCSU was assessed retrospectively over four months using an adapted German version of the Client Sociodemographic and Service Receipt Inventory [32]. Participants were asked about their utilization of inpatient care (general hospitals, psychiatric hospitals and rehabilitation), outpatient physician services (primary care physician, dentist and specialist outpatient physician services, e.g., psychotherapy) and outpatient non-physician services (e.g., physiotherapy, occupational therapy and massage). As the study was based on the baseline sample of a randomized controlled trial, utilization of the intervention was not included to the HCSU.

HrQoL was measured by the Arabic version of the EQ-5D-5L [33]. The questionnaire consists of the five dimensions “mobility”, “self-care”, “usual activities”, “pain/discomfort” and “anxiety/depression”. Each dimension is measured on a scale with the five ordinal response levels “no problems”, “slight problems”, “moderate problems”, “severe problems” and “unable to/extreme problems”. To each of the 5^5^ = 3125 different descriptive health states, EQ-5D-5L index scores have been assigned based on a set of preference valuations of the German general population [34]. The EQ-5D-5L index ranges from −0.66 (extreme problems in all 5 dimensions) to 1.00 (no problems in any dimension). Furthermore, self-rated health was measured by the visual analogue scale of the EQ-5D-5L (EQ-VAS), ranging from 0 (worst imaginable health state) to 100 (best imaginable health state) [35].

PTSS severity was measured by the Arabic version of the PDS-5 [30,36]. The PDS-5 is a self-report questionnaire based on the diagnostic criteria of the DSM-5 that measures PTSS severity on a scale from 0 to 80. A cut-off score of 28 was used to distinguish between persons with a probable PTSD diagnosis (PDS-5 ≥ 28) and persons without a probable PTSD diagnosis (PDS-5 < 28) [30,36].

Furthermore, sociodemographic characteristics on sex, age, educational training level, professional education status, employment status, marital status as well as information with regard to the residence status were collected. Age was categorized into the groups of 18–29, 30–39, 40–49 and ≥50 years. Educational training level was categorized into no graduation, certificate after grade 9/10 (secondary general school), intermediate certificate after grade 10 (secondary school) and higher education entrance qualification after grade 12/13 (academic secondary school). Employment status was categorized into groups of persons working ≥35 h/week, working <35 h/week, being marginally employed and not being employed. The residence status was categorized into asylum entitlement/asylum applicant, refugee protection and other residence permission, namely subsidiary protection, visa and family reunification.

### 2.3. Statistical Analysis

The percentage of missing information across the 65 variables varied between 0% and 17%. In total, 20% of all persons had missing information. It is recommended to impute missing information when missing rates are above 5% [37]. Therefore, missing information in the data was imputed using multiple imputation by chained equations with predictive mean matching to create and analyze 25 multiply imputed datasets [38,39].

Annual health care costs were extrapolated by multiplying 4-month HCSU by the factor 3 and calculated in Euro (EUR) for the year 2019 from a health care payers’ perspective. Patients’ utilization of inpatient care, outpatient physician services and outpatient non-physician services was monetarily valuated using standardized unit costs that were inflated to 2019 price levels using the German gross domestic product inflation rate [40,41]. Annual health care costs and HrQoL were analyzed using descriptive statistics.

Differences in baseline characteristics, HCSU and HrQoL between persons with and without a probable PTSD diagnosis were assessed using linear regressions (F-tests). Associations between health care costs, HrQoL and PTSS severity as well as selected sociodemographic factors were examined using generalized linear models (GLM) with a Poisson family/Gamma family and log link function [42]. These models take into account the skewed distribution of health care costs and HrQoL as dependent variables [43,44]. In order to use the EQ-5D-5L index score as dependent variable in the model, the EQ-5D-5L disutility score (1 − EQ-5D-5L index score) was calculated. As independent variables, the PTSS severity (PDS-5 index) as well as the sociodemographic factors comprising sex, categorized age, educational training level, employment status, marital status and resident status were used.

All analyses were performed using Stata/SE 16.0 (StataCorp, College Station, TX, USA). All applied statistics were two-sided. The level of significance was set at α = 0.05.

## 3. Results

### 3.1. Sample Characteristics

The characteristics of the sample (*n* = 133) are shown in Table 1. The majority of the sample were male (62%; *n* = 82), and the mean age was 33 years. Of the total sample, 39% (*n* = 52) were married or in a partnership, and 61% (*n* = 81) were single, divorced or widowed. More than two thirds of the sample were unemployed (69%; *n* = 92), and 16% of the sample were employed more than marginally (*n* = 21). The majority of the sample had a higher education entrance qualification (71%, *n* = 95). The mean PDS-5-Index score was 28.82, indicating an overall moderate to severe PTSS severity. The mean PDS-5-Index score of persons without probable PTSD diagnosis (*n* = 92) and with probable PTSD diagnosis (*n* = 41) was 17.29 and 38.46, respectively. Furthermore, persons without probable PTSD diagnosis were more often with a higher education entrance qualification compared with persons with probable PTSD diagnosis (78%, *n* = 72 vs. 56%, *n* = 23; *p* = 0.030).

### 3.2. Health Care Services Utilization

In total, 85% (*n* = 113) of the sample utilized health care services within four months (Table 2). The majority of the sample utilized outpatient physician services (84%; *n* = 111), whereas only 11% (*n* = 14) and 13% (*n* = 17) of the sample utilized inpatient care and outpatient non-physician services, respectively. With regard to inpatient care, the mean stays in general hospitals and psychiatric hospitals were 0.43 (standard error (SE) 0.19) and 0.01 (SE 0.01) days per person within four months, respectively, whereas rehabilitation has not been utilized at all. The most commonly utilized outpatient physician services were primary care physicians and dentists with mean contacts of 1.86 (SE 0.23) and 1.42 (SE 0.17) per person within four months, respectively. Specialist outpatient physician services and outpatient non-physician services contacts were on average 1.75 (SE 0.25) and 0.83 (SE 0.27) per person within four months, respectively.

### 3.3. Health Care Costs

The mean total annual health care costs of the sample were EUR 1920 per person (SE EUR 321; Table 2). Of those, the majority of costs were due to outpatient physician services (EUR 1257, SE EUR 129). Inpatient care and outpatient non-physician services accounted for costs of EUR 616 (SE EUR 261) and EUR 47 (SE EUR 14), respectively. Persons with a probable PTSD diagnosis had higher mean total annual health care costs than persons without a probable PTSD diagnosis (EUR 2883, SE EUR 571 vs. 1491, SE EUR 687; *p* = 0.045; Table 3). Thus, the difference in total annual health care costs was mainly due to higher costs incurred by general hospital stays for persons with a probable PTSD diagnosis (EUR 1501, SE EUR 462 vs. 213, SE EUR 309; *p* = 0.020). Mean annual health care costs did not differ statistically significantly between persons with different PTSS severities (Table S1).

The GLM showed that married persons or persons in a partnership had higher mean annual health care costs than persons being single, divorced or widowed (*p* = 0.031; Table 4, model 1). Furthermore, persons aged 40 to 49 had lower mean annual health care costs than persons aged 18 to 29 (*p* = 0.019). Compared with persons with no graduation and no employment, persons with higher education entrance qualification and marginal employment had lower mean annual health care costs (*p* < 0.001 and *p* = 0.010), respectively. Refugee protection was associated with lower mean annual health care costs compared with asylum entitlement and asylum applicant status (*p* = 0.037). Mean total annual health care costs, however, were not associated with PTSD symptom severity measured as PDS-5 index score (*p* = 0.200).

### 3.4. Health-Related Quality of Life

The overall EQ-5D-5L index score and EQ-VAS score of the sample was 0.82 and 73.6, respectively (Table 2). Persons with a probable PTSD diagnosis had a lower EQ-5D-5L index score than persons without a probable PTSD diagnosis (0.71 vs. 0.88; *p* < 0.001; Table 3). The EQ-VAS score did not differ statistically significantly between persons with and without a probable PTSD diagnosis. Mean EQ-5D-5L index scores also differed between persons with different PTSS severities (*p* < 0.001), whereas the EQ-VAS score did not differ statistically significantly (Table S1).

The GLM showed that an increased PDS-5 index score was associated with a higher EQ-5D-5L disutility score (and, therefore, a lower EQ-5D-5L index score; *p* < 0.001; Table 4, model 2). Persons aged 40 to 49 had lower EQ-5D-5L disutility scores than persons aged 18 to 29 (*p* = 0.035). Compared with persons with no graduation, persons with higher education entrance qualification had lower EQ-5D-5L disutility scores (*p =* 0.037).

## 4. Discussion

### 4.1. Main Findings

This study analyzed the economic consequences in terms of HCSU, health care costs and HrQoL in connection with mild to moderate PTSS of Syrian refugees without current psychotherapeutic treatment in Germany based on the “Sanadak” trial. The majority of the study population utilized health care services, predominantly outpatient physician services, within four months. Inpatient care, both in psychiatric hospitals and general hospitals, was utilized only rarely. It is worth mentioning that 15% of the Syrian refugees with mild to moderate PTSS without current treatment did not utilize any health care service within four months. A possible explanation for the low utilization of psychotherapy and psychiatric hospitals might be the exclusion of refugees with severe PTSS/depressive symptoms and the exclusion of refugees with current psychotherapeutic treatment, respectively. In a study concerning disparities in access to health care between asylum seekers and the general population in Germany, 63% of the asylum seekers visited a primary care physician and 25% were admitted to a hospital during 12 months [23]. Furthermore, a lower probability of utilization of outpatient physician services and a higher probability of utilization of outpatient psychotherapeutic treatment and inpatient care for asylum seekers compared with the general population was found. However, higher utilization of outpatient psychotherapeutic treatment by asylum seekers might have been biased, as even though asylum seekers with presumed mental disorders were referred to outpatient psychotherapists, outpatient psychotherapeutic treatment was not successfully started [45]. Therefore, it is possible that the exclusion of persons that were currently in outpatient psychotherapeutic treatment after screening might not have excluded potential health care services users excessively from the analysis (3% of all persons screened). Thus, the low utilization of outpatient psychotherapeutic treatment and psychiatric hospitals might be considered possible. For the current sample of Syrian refugees with mild to moderate PTSS without current treatment, at least the proportion of persons with utilization of general hospitals within four months was comparable (11%), whereas the proportion of persons with utilization of primary care physicians was considerably higher already within four months (84%).

The annual health care costs resulting from the HCSU of the Syrian refugees with mild to moderate PTSS without current treatment of around EUR 2000 per person seemed to be relatively low compared with the annual health care costs for persons with PTSD in Germany ranging from more than EUR 9000 to almost EUR 15,000 [21,46]. Even for those refugees with a probable PTSD diagnosis, the annual health care costs of around EUR 3000 were distinctly below those costs. One explanation for this might be that in the current study, the total annual health care costs comprised only costs for inpatient care, outpatient physician services and outpatient non-physician services. It is worth noting that the majority of the difference in total health care costs between refugees with and without a probable PTSD diagnosis was due to statistically significantly higher costs for general hospital stays.

### 4.2. Previous Research and Possible Explanations

In a study analyzing the economic consequences of PTSD in adolescents and young adults in Germany, half of the total health care costs were attributable to psychiatric hospitals, general hospitals and rehabilitation [46]. In contrast, in the current sample, costs attributable to inpatient care were only one third of the total health care costs. The low HCSU and the low health care costs, especially related to psychiatric hospitals, might be an indication for insufficient or even inappropriate health care with respect to the PTSS. However, results of the current study may not be directly comparable to those of the aforementioned study, as the sample there was younger and recruited in clinical settings after a clinically definite diagnosis of PTSD [46].

HCSU and thus health care costs are determined by predisposing sociodemographic characteristics, such as age, sex, marital status, educational training and employment [47]. In the current study, those determinants of health care costs, with the exception of sex, could be confirmed for Syrian refugees with PTSS. However, against expectations, persons aged 40 to 49 had lower annual health care costs compared with persons aged 18 to 29. With respect to employment as predisposing factor, it is noticeable that for marginally employed persons, annual health care costs were lower compared with annual health care costs of unemployed persons. Furthermore, the residential status of the refugees determined annual health care costs. Residential status of refugees, especially refugee protection, can also be seen as an enabling determinant of HCSU [47]. Contrary to our expectations, more severe PTSS, which can be considered a need factor determining HCSU, were not associated with higher annual health care costs [48].

The HrQoL of the Syrian refugees with PTSS was lower compared with the HrQoL of a representative German population sample (0.82 vs. 0.88) [49]. However, for those refugees without a probable PTSD diagnosis, the HrQoL did not differ compared with the HrQoL of the German general population. Comparability of the representative German population sample with persons of the current study is limited, as the persons of the German population sample were older, and fewer persons were men [49]. In another study from Germany, the mental HrQoL of asylum applicants and refugees was higher than the mental HrQoL of the German general population, whereas the physical HrQoL was lower [18,50,51]. To the best of our knowledge, no studies that assessed the HrQoL of Syrian populations were available. However, a study from Sweden that assessed the HrQoL of Syrian refugees found a lower HrQoL than the current study (0.75 vs. 0.82) [52]. Nevertheless, it remains unclear whether the differences in HrQoL between those populations were either due to differences in PTSS severity, migration as a potential determinant of HrQoL or intercultural differences in the perception of the own health and HrQoL.

HrQoL of Syrian refugees was mainly determined by severity of PTSS. Furthermore, those aged 40 to 49 and those without graduation had a lower HrQoL compared with refugees aged 18 to 29 and refugees with higher education entrance qualification, respectively. In a study of HrQoL of asylum seekers and refugees in Germany, a lower HrQoL was associated with female sex, older age, unemployment and having never been married, separated or divorced [18]. In the current study, no association between HrQoL, sex and marital status was found. However, direct comparison with the aforementioned study is limited, as the sample consisted also of asylum seekers and refugees from other countries of origin that differed significantly in their HrQoL [18].

### 4.3. Implications for Health Care Services and Policy

With respect to adequate provision of health care services for Syrian refugees with PTSS, it is crucial to identify those refugees in need for health care and to guide those through the process of seeking health care. The low annual health care costs of Syrian refugees with mild to moderate PTSS without current treatment could be an indication for underprovision of health care services. Such underprovision might be induced by barriers to access health care services, such as language barriers, mental health stigma, or lack of awareness of asylum seekers and refugees [53,54]. Eventually, the provision of effective evidence-based care may reduce PTSS and subsequently improve HrQoL. Furthermore, provision of target-group-specific and low-threshold accesses to health care may contribute to adequate provision of health care services, particularly for Syrian refugees with PTSS. Finally, promotion of access to education and employment might have a positive effect on HrQoL of refugees in general.

### 4.4. Strengths and Limitations

The major strength of this study was the multistrategic approach to recruiting such a hard-to-reach population of Syrian refugees with PTSS [28]. Furthermore, study participants had access to assessment instruments available in the Arabic language. HCSU was assessed using a service receipt inventory that has been proven effective in practical application for the evaluation of cost of health care in Germany. Furthermore, missing information across the variables used for the analyses was imputed using a state-of-the-art imputation strategy.

However, there are also limitations to consider. HCSU was assessed retrospectively over four months using self-assessment and extrapolated to annual health care costs. Furthermore, the service receipt inventory used for assessment of HCSU was shortened. Information on utilization of medication, formal and informal care, as well as on absenteeism from work owing to illness and reduced productivity during work was not collected. However, it was assumed that at least the utilization of formal and informal care would be negligible for the assessment of HCSU of Syrian refugees with PTSS. Last, as this study was based on the baseline sample of a randomized controlled trial, generalizability was limited to Syrian refugees with mild to moderate PTSS severity without current treatment. Exclusion of five persons that were currently in outpatient psychotherapeutic treatment might have led to an underestimation of HSCU and health care costs. Furthermore, exclusion of persons with severe depressive symptoms and pregnant persons might have reduced external validity and distorted HSCU and cost estimates. However, no more than five persons (3% of all persons screened) were excluded after screening due to severe depressive symptoms, pregnancy and illiteracy.

## 5. Conclusions

Among Syrian refugees with mild to moderate PTSS without current treatment that arrived in Germany during the European migrant and refugee crisis, HCSU and the resulting health care costs are low. HrQoL in this group was reduced compared with HrQoL of other refugee populations or the German general population without PTSS. Furthermore, more severe PTSS were negatively associated with HrQoL.

## Figures and Tables

**Table 1:** Sample characteristics (*n* = 133).

Characteristics	Total Sample (*n* = 133)	Without Probable PTSD Diagnosis (PDS-5-Index < 28, *n* = 92)	With Probable PTSD Diagnosis (PDS-5-Index ≥ 28, *n* = 41)
Age: mean years (SE)	33.34 (0.97)	33.10 (1.12)	33.88 (1.92)
Female sex: *n* (%)	51 (38.35)	33 (35.87)	18 (43.90)
Marital status: *n* (%)			
Single/divorced/widowed	81 (61.08)	56 (61.30)	25 (60.59)
Married/in partnership	52 (38.92)	36 (38.70)	16 (39.41)
Educational training: *n* (%)			
No graduation	16 (12.06)	8 (8.74) *	8 (19.51) *
Certificate after grade 9/10	18 (13.62)	8 (8.83)	10 (24.39)
Intermediate certificate (after grade 10)	4 (3.10)	4 (4.48)	0 (0.00)
Higher education entrance qualification (after grade 12/13)	95 (71.22)	72 (77.96)	23 (56.10)
Professional education: *n* (%)			
No professional qualification	69 (51.58)	44 (48.35)	25 (58.83)
Professional qualification	7 (5.32)	4 (3.87)	3 (8.59)
University degree ^†^	57 (43.10)	44 (47.78)	13 (32.59)
Employment: *n* (%)			
Working ≥35 h/week	8 (6.02)	7 (7.61)	10 (7.44)
Working <35 h/week	13 (9.77)	9 (9.78)	40 (9.76)
Marginal employment	20 (15.04)	17 (18.48)	30 (7.32)
Not employed	92 (69.17)	59 (64.13)	33 (80.49)
Resident status: n (%)			
Asylum entitlement/applicant	74 (55.64)	52 (56.52)	22 (53.66)
Refugee protection	28 (21.05)	17 (18.48)	11 (26.83)
Other residence permission ^‡^	31 (23.31)	23 (25.00)	8 (19.51)
Years in Germany: mean (SE)	3.05 (0.11)	2.99 (0.12)	3.17 (0.21)
PDS-5-Index: mean (SE)	28.82 (1.01)	17.29 (0.55) ***	38.46 (1.23) ***

Comments: SE: standard error, PDS-5: Posttraumatic Diagnostic Scale for DSM-5; comparisons of sample characteristics by post-traumatic stress disorder (PTSD) diagnosis were analyzed using the F-test; ^†^ Including polytechnic degree, technician and master craftsmen; ^‡^ Subsidiary protection, visa, family reunification; * *p* ≤ 0.05, *** *p* ≤ 0.001.

**Table 2:** The 4-month health care services utilization, annual health care costs and health-related quality of life of refugees with PTSD symptoms (*n* = 133).

Cost Category/Measure of Health Effect	% of the Sample with Utilization	Mean Utilization in Days/Contacts (SE)	Mean Annual Costs/Value	Median Annual Costs/Value	SE of Annual Costs/Value	Annual Cost/Value Range
Inpatient care	10.53	0.44 (0.19)	€616	€0	€261	€0 to €31,732
General hospital	10.53	0.43 (0.19)	€610	€0	€261	€0 to €31,732
Psychiatric hospital	0.75	0.01 (0.01)	€6	€0	€6	€0 to €790
Outpatient physician services	83.46	5.02 (0.48)	€1257	€748	€129	€0 to €6963
Primary care physician	57.14	1.86 (0.23)	€124	€67	€15	€0 to €1005
Dentist	51.88	1.42 (0.17)	€244	€173	€30	€0 to €1729
Specialists	48.12	1.75 (0.25)	€888	€436	€101	€0 to €5174
Outpatient non-physician services	12.78	0.83 (0.27)	€47	€0	€14	€0 to €1330
Total	84.96	-	€1920	€975	€321	€0 to €36,651
EQ-5D-5L-Index	-	-	0.82	0.88	0.02	0.07 to 1.00
EQ-VAS	-	-	73.61	70.00	1.64	10 to 100

Comments: SE: standard error, VAS: Visual Analogue Scale.

**Table 3:** Mean annual health care costs and health-related quality of life of refugees with and without a probable PTSD diagnosis (*n* = 133).

Cost Category/Measure of Health Effect	Without Probable PTSD Diagnosis (PDS-5-Index < 28, *n* = 92)	With Probable PTSD Diagnosis (PDS-5-Index ≥ 28, *n* = 41)	Difference		
	Mean (SE)	Mean (SE)	Mean (SE)	95% CI	*p*-Value ^&^
Inpatient care	€213 (€309)	€1521 (€463)	€1308 (€557)	€206; €2410	0.020 *
General hospital	€213 (€309)	€1501 (€462)	€1289 (€556)	€188; €2389	0.022 *
Psychiatric hospital	€0 (€0)	€19 (€19)	€19 (€13)	−€6; €45	0.135
Outpatient physician services	€1249 (€156)	€1275 (€234)	€26 (€281)	−€530; €582	0.926
Primary care physician	€112 (€18)	€152 (€27)	€40 (€33)	−€25; €105	0.226
Dentist	€280 (€36)	€164 (€53)	−€116 (€64)	−€243; €12	0.075
Specialists	€857 (€121)	€958 (€182)	€102 (€219)	−€331; €535	0.643
Outpatient non-physician services	€29 (€18)	€88 (€26)	€59 (€32)	−€4; €122	0.068
Total costs	€1491 (€381)	€2883 (€571)	€1393 (€687)	€33; €2752	0.045 *
EQ-5D-5L-Index	0.88 (0.02)	0.71 (0.03)	−0.17 (0.03)	−0.23; −0.11	<0.001 ***
EQ-VAS	74.14 (1.99)	72.32 (2.96)	−1.87 (3.55)	−8.90; 5.15	0.609

Comments: SE: Standard error, VAS: Visual Analogue Scale, PDS-5: Posttraumatic Diagnostic Scale for DSM-5. ^&^ Based on F-test; * *p* ≤ 0.05, *** *p* ≤ 0.001.

**Table 4:** Influence of selected sociodemographic characteristics on annual health care costs and EQ-5D-5L disutility scores using generalized linear models with robust standard errors (*n* = 133).

Independent Variables	Dependent Variable					
Sociodemographic Characteristics	Annual Health Care Costs (Model 1)			EQ-5D-5L Disutility Scores ^‡^ (Model 2)		
	Beta (SE)	95% CI	*p*-Value	Beta (SE)	95% CI	*p*-Value
PDS-5 Index	0.02 (0.02)	−0.01; 0.05	0.200	0.05 (0.01)	0.03; 0.06	<0.001
Gender (ref. male)						
Female	−0.37 (0.35)	−1.05; 0.30	0.279	0.18 (0.20)	−0.20; 0.56	0.353
Age category (ref. [18,19,20,21,22,23,24,25,26,27,28,29])						
30–39	−0.24 (0.37)	−0.97; 0.48	0.507	−0.12 (0.22)	−0.55; 0.32	0.146
40–49	−0.87 (0.37)	−1.59; −0.14	0.019	−0.70 (0.33)	−1.35; −0.05	0.035
≥50	−0.32 (0.49)	−0.64; 1.28	0.517	0.05 (0.25)	−0.45; 0.55	0.839
Education (ref. no graduation)						
Certificate after grade 9/10	−0.94 (0.53)	−1.97; 0.10	0.077	−0.83 (0.43)	−0.67; 0.01	0.054
Intermediate certificate (after grade 10)	−0.53 (0.51)	−1.54; 0.47	0.300	−0.65 (0.56)	−0.75; 0.45	0.246
Higher education entrance qualification (after grade 12/13)	−0.93 (0.25)	−1.41; −0.44	<0.001	−0.68 (0.32)	−1.31; 0.04	0.037
Employment (ref. not employed)						
Marginal employment	−0.81 (0.32)	−1.43; −0.19	0.010	−0.34 (0.24)	−0.81; 0.12	0.150
Working <35 h/week	−0.49 (0.40)	−1.28; 0.28	0.223	−0.65 (0.56)	−0.70; 0.21	0.296
Working ≥35 h/week	−0.58 (0.48)	−1.52; 0.35	0.220	−0.68 (0.32)	−1.10; 0.19	0.171
Marital status (ref. single/divorced/widowed)						
Married/in partnership	0.73 (0.34)	0.07; 1.39	0.031	−0.23 (0.24)	−0.71; 0.24	0.331
Resident status (ref. asylum entitlement/applicant)						
Refugee protection	−0.63 (0.30)	−1.22; −0.04	0.037	−0.48 (0.26)	−0.98; 0.03	0.063
Other residence permission ^†^	0.08 (0.32)	−0.71; 0.54	0.795	0.16 (0.23)	−0.29; 0.61	0.493
Constant	7.99 (0.47)	7.07; 8.91	<0.001	−2.18 (0.39)	−2.95; −1.42	<0.001

Comments: CI: confidence interval, PDS-5: Posttraumatic Diagnostic Scale for DSM-5; ^†^ Subsidiary protection, visa, family reunification; ^‡^ 1 − EQ-5D-5L index scores.

## Data Availability

The data presented in this study are available on reasonable request from the corresponding author.

## Supplementary Materials

The following are available online at https://www.mdpi.com/1660-4601/18/7/3408/s1; Table S1: Mean annual health care costs and health-related quality of life of refugees with different post-traumatic stress symptom severities based on the PDS-5-Index (*n* = 133).
