# Supplementary material for: Health Care Services Utilization and Health-Related Quality of Life of Syrian Refugees with Post-Traumatic Stress Symptoms in Germany (the Sanadak Trial)
Source: Int J Environ Res Public Health. 2021 Mar 25;18(7):3408. doi: 10.3390/ijerph18073408 (PMC8036810; doi:10.3390/ijerph18073408)
Supplement: Supplementary file 1 [file ijerph-18-03408-s001.pdf]

Table S1: Mean annual health care costs and health-related quality of life of refugees with different post-traumatic stress symptom severities based on the PDS-5-Index † (*n* = 133).

Cost Category/Measure of health Effect	Mild Symptoms (*n* = 8)	Moderate Symptoms (*n* = 56)	Moderate to Severe Symptoms (*n* = 45)	Severe Symptoms (*n* = 24)	*p*-Value &
	**Mean (SE)**	**Mean (SE)**	**Mean (SE)**	**Mean (SE)**	
Inpatient care	€0 (€1057)	€274 (€399)	€483 (€446)	€1869 (€610)	0.152
General hospital	€0 (€1055)	€274 (€399)	€465 (€445)	€1869 (€609)	0.149
Psychiatric hospital	€0 (€24)	€0 (€9)	€18 (€26)	€0 (€14)	0.587
Outpatient physician services	€816 (€530)	€1302 (€200)	€1148 (€223)	€1503 (€305)	0.654
Primary care physician	€92 (€61)	€101 (€23)	€121 (€26)	€198 (€35)	0.131
Dentist	€259 (€122)	€281 (€46)	€242 (€52)	€158 (€71)	0.550
Specialists	€464 (€411)	€920 (€155)	€785 (€173)	€1146 (€237)	0.454
Outpatient non-physician services	€21 (€61)	€35 (€23)	€43 (€26)	€92 (€35)	0.554
Total	€836 (€1296)	€1611 (€490)	€1674 (€547)	€3463 (€748)	0.141
EQ-5D-5L-Index	0.91 (0.06)	0.91 (0.02)	0.81 (0.02)	0.64 (0.03)	<0.001 ***
EQ-VAS	75.63 (6.68)	76.72 (2.56)	71.22 (2.82)	70.00 (3.85)	0.367

Comments: SE: Standard error, VAS: Visual Analogue Scale, PDS-5: Posttraumatic Diagnostic Scale for DSM-5
& Based on F-test; † Mild PTSD symptoms: 1 ≤ PDS-5-Index < 11, moderate PTSD symptoms: 11 ≤ PDS-5-Index < 21, moderate to severe PTSD symptoms: 21 ≤ PDS-5-Index < 36, severe PTSD symptoms: PDS-5-Index ≥ 36; * $p \leq 0.05$, ** $p \leq 0.01$, *** $p \leq 0.001$
